# Associations between the 3D position of the mental foramen with sagittal skeletal relationships (classes I, II, and III) and vertical facial growth patterns (normal, long, and short faces) in different ages and sexes: a retrospective cohort study of 360 CBCTs

**DOI:** 10.1186/s12903-023-03719-z

**Published:** 2023-12-05

**Authors:** Sepideh Bagheri, Mohammadreza Shokuhifar, Mehrnaz Moradinejad, Mahshid Razavi, Alireza Hashemi Ashtiani, Behnaz Baratvand, Vahid Rakhshan

**Affiliations:** 1 Department of Orthodontics, School of Dentistry, Ahvaz Jundishapur University of Medical Sciences, Ahvaz, Iran; 2 Department of Oral & Maxillofacial Radiology, School of Dentistry, Ahvaz Jundishapur University of Medical Sciences, Ahvaz, Iran; 3 Department of Prosthodontics, School of Dentistry, Ahvaz Jundishapur University of Medical Sciences, Ahvaz, Iran; 4 Department of Anatomy, Azad University of Medical Sciences, Tehran, Iran

**Keywords:** Surgery, Implantology, Anatomy, Mental Foramen, Orthodontics, 3D position, Skeletal Malocclusion (classification), Vertical Growth patterns, Age, Sex dimorphism

## Abstract

**Background:**

The 3D position of the mental foramen (MF) is of significant clinical value in dental implantology and mandibular surgeries or in local anesthesia. Despite its importance, it is not clearly known how the position of MF can alter in different individuals, since the literature on the associations between the MF position with vertical growth patterns is non-existent and those on links between the MF position and skeletal malocclusions are scarce. Therefore, we aimed to investigate these, for the first time, on cone-beam computed tomographies (CBCTs).

**Methods:**

Archival CBCTs of 9 sub-groups (i.e., 3 skeletal Classes I, II, and III × 3 vertical growth patterns ‘long face, short face, normal face’) were collected by evaluating patients’ SNA, SNB, ANB, facial angle, lower facial height, and FMA (n = 9 × 40 = 360). Included cases were older than 17 years and without any history of orthodontic/orthognathic treatments (243 women, 117 men, mean age: 22.28 ± 2.80 years). Perpendicular distances between the MF and 3 fixed bony structures (the mandibular symphysis [S/width], the mandibular ramus [R/length], and the mandibular lower cortex [C/height]) were measured on different sectional planes on both hemimandibles. Left- and right-side measurements were combined. Data were analyzed using the 3-way ANCOVA, Bonferroni, one-way ANOVA, Tamhane, Pearson, and t-test (α = 0.05).

**Results:**

Width was the smallest in Class II and greatest in Class III cases (all *P* values < 0.000001, Bonferroni). It was the shortest in long faces and longest in short faces (all *P* values ≤ 0.00008). The inferior-superior height was larger in Class III than both Classes I and II (both *P* values ≤ 0.003); there was no significant difference between Classes I and II in terms of height (*P* = 0.684). Height was the largest in long faces and smallest in short faces (all *P* values < 0.000001). The anterior-posterior length was the largest in Class III and smallest in Class II (all *P* values < 0.000001). Length was larger in short-face people versus normal- or long-face individuals (*P* ≤ 0.00003); nevertheless, long and normal faces did not differ in terms of length (*P* = 0.448). Subjects’ age was not correlated with their MF positions (*P* ≥ 0.579, Pearson coefficient). Sex dimorphism existed only for height (*P* = 0.009, t-test) but not for length or width.

**Conclusions:**

The MF position may considerably differ in various horizontal or vertical growth patterns and sexes. This should be noted in mandible surgeries.

## Introduction

Inserting dental implants and performing other surgical operations as well as the administration of local anesthesia in a great area of the mandible need utmost care regarding the safety of the mental foramen (MF) [1–3]. The MF is a bilateral opening on the anterior surface of the mandible, from which the terminal branch of the inferior alveolar nerve (mental nerve) and blood vessels protrude [1–3]. The mental nerve unilaterally provides sensory innervation for the lower lip, the labial mucosa, the lower canine, and the lower premolars [4]. Since the mandible is constantly growing, the exact location of the MF depends on age, sex, ethnic origin, shape, size, and symmetry of the skull and facial structures [1–3, 5, 6]. For example, in the elderly, due to the atrophic mandible, the mental foramen shifts upward, while in children, it is lower and closer to the inferior border of the mandible [7].

Since the MF is not clinically palpable and its location varies on the buccal surface of the mandibular body, the knowledge of its exact location, shape, size, and number of MFs is essential for various clinical dental procedures [8]. Accurate locating of the MF is vital for proper treatment planning, regional anesthesia, deciding about the incision length and location, or decision on the flap height, osteotomy, and implant placement; accordingly, the awareness of its site is key to preventing damage, either transient or irreversible, to the neurovascular bundle [1, 8, 9].

Maxillofacial growth patterns may affect the treatment planning because variations across individuals alter the position of some anatomical structures. In this regard, faces are categorized in the vertical and horizontal dimensions. Through the vertical dimension, facial growth patterns are described as long, normal, and short faces [10]. The horizontally categorized patterns on the sagittal plane comprise the Class I (normal), Class II, and Class III [1, 11].

In various studies, two-dimensional (2D) radiographic techniques such as panoramic [12] and cephalometric [13] radiograms have been used to determine the location of the MF. Due to the limited overlap of structures in 2D radiographic techniques, they cannot accurately determine the MF’s position. For this reason, a radiographic method using three-dimensional (3D) technology is more valuable than the 2D radiographic techniques [8, 14, 15]. Cone-beam computed tomography (CBCT) is a 3D radiographic approach that overcomes the limitations of conventional radiography by producing 3D images [16]. CBCT is helpful in providing complete information about the jaw and face structure and thus can be used to assess anatomical features and identify any pathologies [17].

Studying the MF anatomy is valuable: Knowing the correct position of the MF and its anatomical variations is important in many dental surgeries. The position of mental foramen can differ across various ethnic groups [18], marking the importance of conducting studies on this matter in different populations. Besides, the existing studies on this regard are mostly controversial and lack a large sample [19–23]. More importantly, the literature regarding the associations between the anatomy of mental foramen with horizontal or vertical facial growth patterns is quite scarce; they are also limited by methodological shortcomings, rather small samples, and/or a rather narrow span of the assessed variables [1]. Finally, to the best of our knowledge, there is no study on the link between the position of the MF and the vertical growth pattern of the person (i.e., long face, short face, and normal face). Therefore, this two-way balanced CBCT study aimed to examine the associations between the position of the mental foramen with different vertical growth patterns and horizontal skeletal Classes.

## Materials and methods

### Patients and study design

This large, balanced retrospective cohort study was performed on the archival CBCTs of 360 patients referred to the radiology department of Ahvaz Jundishapur University of Medical Sciences: 3 skeletal malocclusion groups, each including 120 patients – also at the same time, 3 vertical growth patterns, each including 120 patients.

All the CBCTs were archival and no patient was exposed to any X-ray due to this study. No personal information was collected. Therefore, no patient was harmed by this study. Since this study was performed on retrospectively taken anonymized human data, the need for informed consent to participate was waived by the Institutional Review Board of Ahvaz Jundishapur University of Medical Sciences, Ahvaz, Iran (ethics number: IR.AJUMS.REC.1400.120). This retrospective cohort study and its ethics were approved by the Ethics Committee of Ahvaz Jundishapur University of Medical Sciences (ethics code: IR.AJUMS.REC.1400.120). All methods were performed in accordance with the relevant guidelines and regulations (including the Declaration of Helsinki).

### Eligibility criteria

The inclusion criteria for selecting patients were as follows: the mental foramen should be visible, the presence of the mandibular teeth at least up to the first molar, the teeth were fully erupted and without severe caries, and without any history of any orthodontic treatment. Excluded were patients younger than 18 years of age with crowding, spacing between the teeth, or any orthodontic appliances. Also excluded were cases with the presence of a fracture or lesion that might prevent an accurate diagnosis of the mental foramen, and the presence of bone resorption around the teeth.

### Sample size

Since there was no similar study to use for power calculations, we aimed to collect a sample much larger than previous (remotely similar) studies. The sample size was pre-determined as greater than 2.5 times the size of a 2022 study that had some similarities to this research [1].

### Data curation

The sample was collected consecutively from the archives until acquiring 360 cases in a two-way balanced way, i.e., the sampling procedure continued until acquiring 9 sub-groups of 40 each, consisting of 9 various combinations of the 3 skeletal Classes and the 3 vertical growth patterns. In other words, sampling was carried out until acquiring 120 subjects in each of the 3 skeletal malocclusions (Classes I, II, and III) and simultaneously until obtaining 120 subjects in each of the 3 vertical growth patterns (short face, normal face, and long face). For finding and diagnosing these cases, cephalogram constructs of CBCTs were created for each patient; then, certain cephalometric measurements were measured on them (detailed below). Each of the skeletal malocclusion groups ‘the Class I, Class II, and Class III’ (n of each = 120) would include the same number of ‘short face, normal face, and long face’ subjects (n of each of the 9 sub-groups = 40). At the same time, each of the groups ‘short face, normal face, and long face’ would have the same number of patients from Classes I, II, and III (n of each of the 9 sub-groups = 40).

### CBCT settings

A CBCT device (NewTom VGi, QR, and Verona, Italy) with a field of view (FOV) of 15 × 15 was used. All scans were taken with the exposure conditions of 110 KVp and 7.2 MA and with a voxel size of 0.300 mm^2^. All patients were in the upright position and instructed not to swallow or move their head or tongue while scanning.

All scans were analyzed using specialized computer software (NNT viewer v9, QR, Verona, Italy). Simulated lateral cephalographs were created from CBCT volumes. Images were viewed in a semi-dark room on a monitor screen (LED, flat screen, 14 inch) with a 1920 × 1080 resolution.

### Cephalometric analysis

An orthodontist performed all cephalometric analyses. To determine the skeletal Classes of patients as well as their vertical growth patterns, the following analyses and measurements were used: Down analysis (including the SNA, SNB, ANB angles [the angles between sella, nasion, A point, and B point]), Tweed analysis (including the mandibular plane angle and the FMA [Frankfurt-Mandibular plane angle]), and Ricketts analysis (including the facial angle [the angle between the N-Pog line and the Frankfurt plane] and the lower facial height [the distance between the anterior nasal spine and menton]) [24]. The cephalometric variables SNA, SNB, ANB, and the facial angle were used to determine the horizontal skeletal Classes, while FMA and the lower facial height were used to determine the vertical growth patterns [24]. The normal ranges for the evaluated cephalometric variables were as follows: SNA: 80 ± 2 °, SNB: 78 ± 2 °, ANB: 0–2 °, facial angle: 87–93 °, lower facial height: 44–46 mm, and FMA: 20–30 ° [24].

### The 3D position of the mental foramen

Two trained observers (blinded to the cephalometric measurements and the skeletal malocclusions and vertical growth patterns of patients) jointly analyzed multiplanar CBCT reconstructions to identify the mental foramen on both the left and right hemimandibles. Three parameters were used to determine and quantize the 3D position of the MF with respect to the fixed bony structures around it.

#### S

**Fig. 1:**
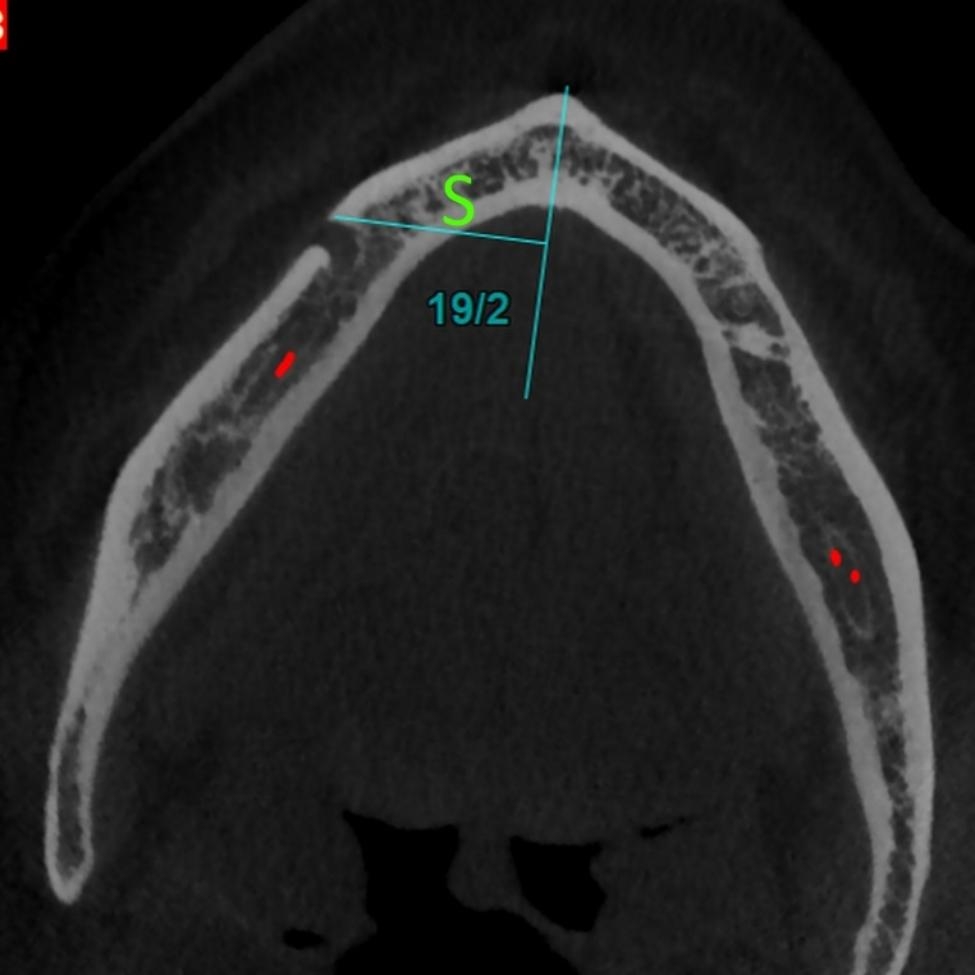
The parameter S (width) or the perpendicular distance to the symphysis on the axial plane. This distance shows the horizontal “width” dimension

The perpendicular distance to the mandibular symphysis on the axial plane (Fig. 1). This distance shows the horizontal “width” dimension.

#### C

**Fig. 2:**
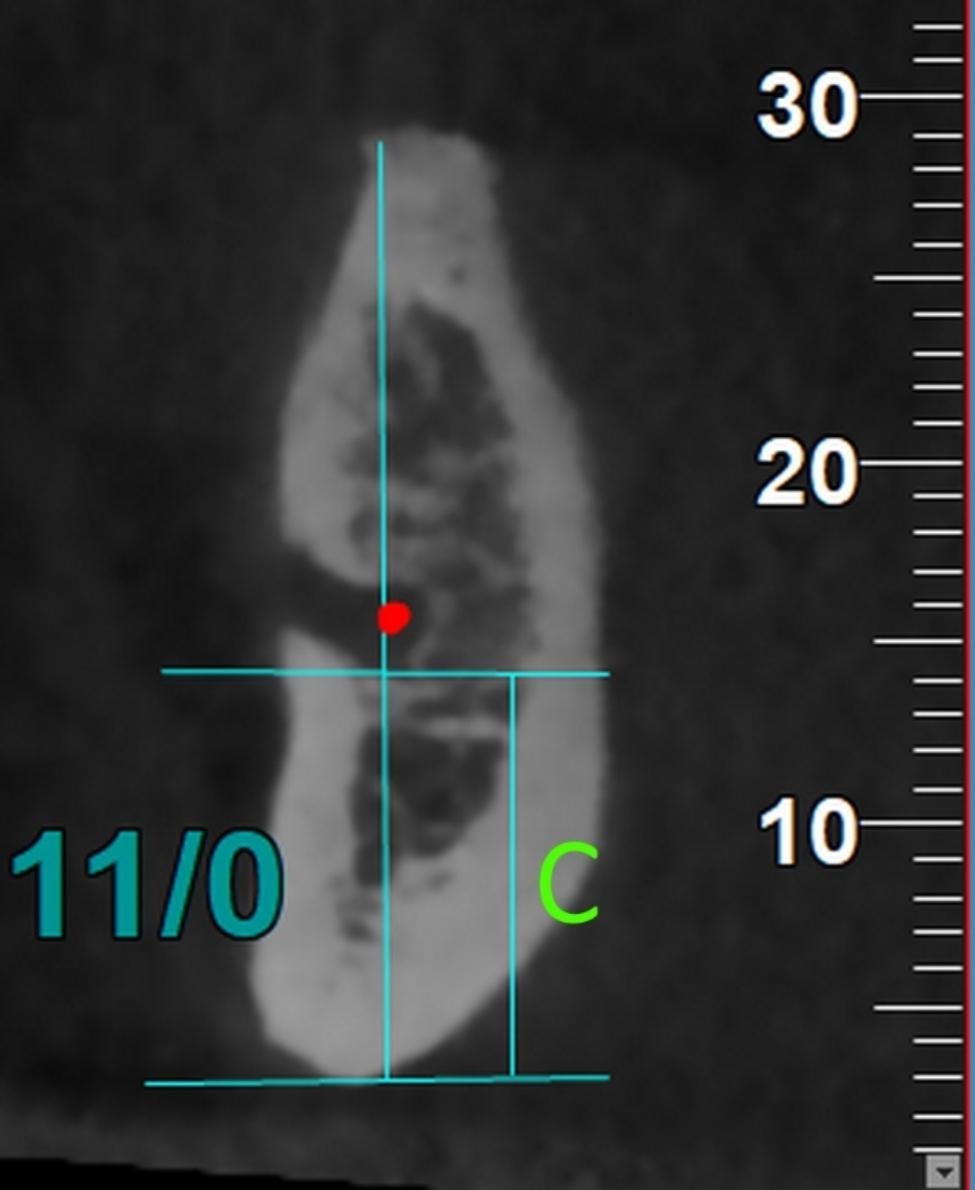
The parameter C (height) or the perpendicular distance to the inferior cortex of the mandible on the cross-sectional plane. This distance represents the inferior-superior “height” dimension

The perpendicular distance to the inferior cortex of the mandible on the cross-sectional plane (Fig. 2). This distance represents the inferior-superior “height” dimension.

#### R

**Fig. 3:**
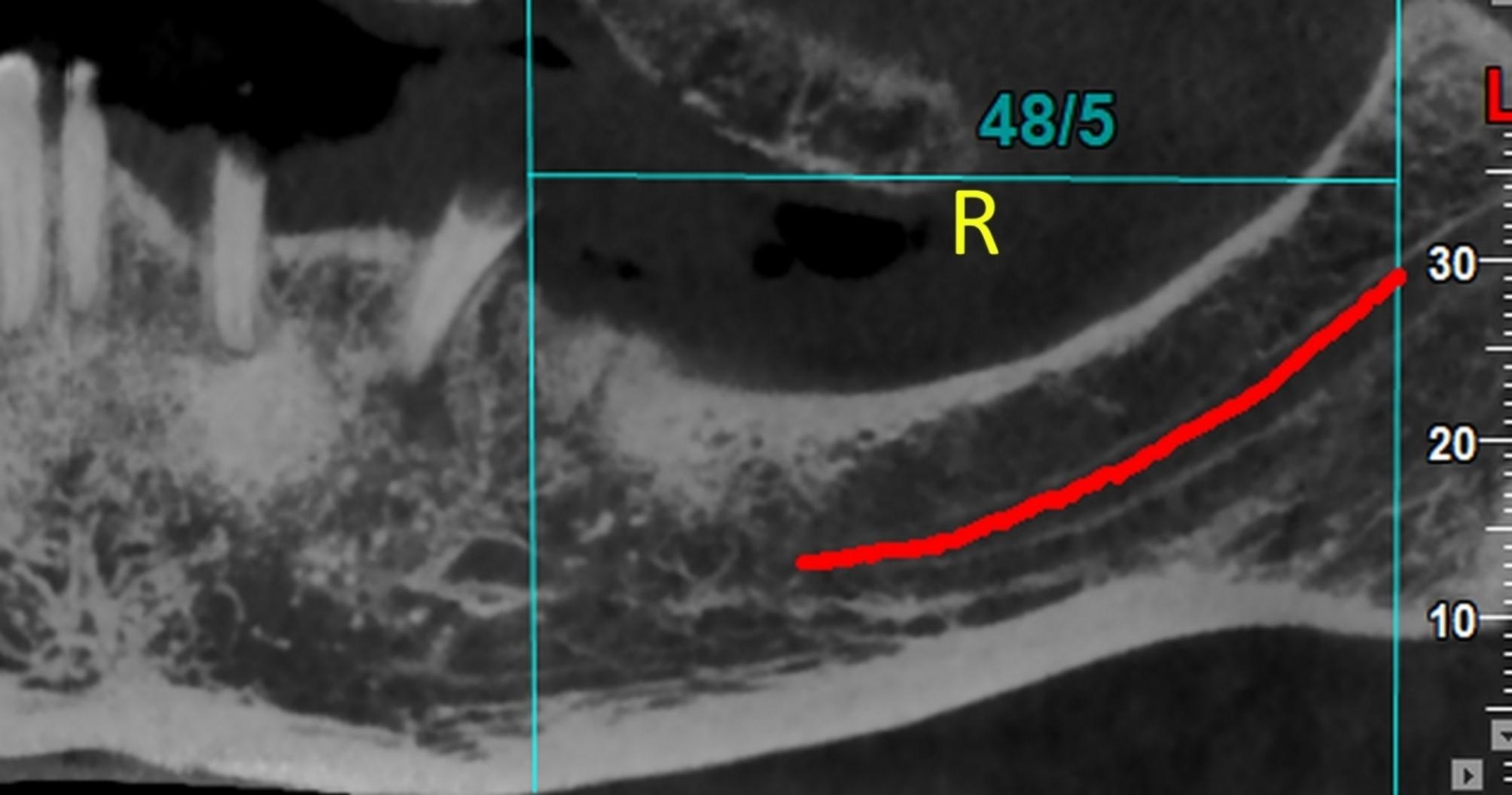
The parameter R (length) or the perpendicular distance to the anterior border of the ramus on the parasagittal plane. This distance indicates the anterior-posterior “length” dimension

The perpendicular distance to the anterior border of the ramus on the parasagittal plane (Fig. 3). This distance indicates the anterior-posterior “length” dimension.

Most patients had both left and right mental foramens. Few patients had only one mental foramen. If a patient had both the left and right mental foramina, for each of the above 3 parameters of the left and right mental foramens, the average of both foramens would be calculated for that patient. If a patient had only 1 mental foramen (and lacking the second one), for each of the above 3 parameters, only 1 value would be recorded for that patient and there would be no averaging.

### Intraobserver agreement

To calculate the intraobserver agreement, one of the observers randomly selected 4 cases from each of the 9 subgroups (n = 36), and measured the variables C, R, and S for mental foramens again. A Cronbach Alpha was calculated for each of the variables to examine the intraobserver agreement. The intraobserver agreement for the S parameter was perfect (Cronbach Alpha = 0.990, *P* < 0.000001); so were the intraobserver agreements for C (Cronbach Alpha = 0.988, *P* < 0.000001) and R (Cronbach Alpha = 0.979, *P* < 0.000001).

### Statistical analysis

Descriptive data and 95% confidence intervals (CI) were calculated. The groups were compared using the following tests: a 3-way analysis of covariance (ANCOVA) followed by a Bonferroni post hoc test, a 1-way analysis of variance (ANOVA) followed by a Tamhane post hoc test, a chi-squared test, and an independent-samples t-test. Also, a Pearson correlation coefficient was used to test the link between age and the 3D position of the MF. The software in use was SPSS 25 (IBM, Armonk, NY, USA). The level of significance was set at 0.05.

## Results

A total of 1253 CBCTs were evaluated against the eligibility criteria until finding 9 sub-groups of 40 cases each (360 cases). There were no missing data. There were 243 women and 117 men with a mean age of 22.28 ± 2.80 years. The mean (SD) age of patients of skeletal Classes I, II, and III were 22.27 ± 2.89, 22.28 ± 2.72, and 22.31 ± 2.81 years, respectively (min = 18, max = 28 for each of the 3 groups). There was not a significant difference between the ages of these 3 groups (t-test, *P* = 0.993). The mean (SD) age of patients in vertical growth patterns ‘short face, normal face, and long face’ were respectively 22.35 ± 3.09, 22.22 ± 2.57, and 22.28 ± 2.75 years (min = 18, max = 28 for each of the 3 groups). There was not a significant difference between the ages of these 3 groups either (t-test, *P* = 0.935).

In vertical growth patterns ‘short face, normal face, and long face’, there were 91, 80, and 72 women, respectively. In these groups, there were 29, 40, and 48 men, respectively. The chi-square test showed a slight but statistically significant difference between the distributions of sexes across these 3 groups (*P* = 0.032). In skeletal Classes I, II, and III there were respectively 85, 81, and 77 women, and 35, 39, and 43 men. The sexes were similarly distributed across these 3 Classes (*P* = 0.545, chi-square).

### Mandibular plane angle

In vertical growth groups ‘short face, normal face, and long face’, there were respectively 79, 13, and 0 cases with low mandibular plane angles, 41, 60, 44 cases with normal mandibular plane angles, and 0, 47, and 76 cases with high mandibular plane angles. The distributions of mandibular plane angles differed significantly across the groups ‘short face, normal face, and long face’ (*P* = 0.000, chi-square). The mandibular plane angles in horizontal skeletal Classes were as follows: In Classes I, II, and III, there were respectively 33, 40, and 19 low angles, 52, 47, and 46 normal angles, and 35, 33, and 55 high angles. These mandibular angle distributions were significantly different among skeletal Classes I, II, and III (*P* = 0.004).

### Cephalometrics

Descriptive statistics and 95% CIs for the cephalometric variables as well as the results of the one-way ANOVA comparisons across different skeletal Classes and also among different vertical growth patterns are presented as Tables 1 and 2. The Tamhane post hoc test showed that all the pairwise comparisons performed after the significant ANOVAs were significant (all *P* values < 0.000001). The ANOVA showed that only FMA and the lower facial height were not significantly different across Classes I, II, and III (Table 1). The Tamhane post hoc test showed that all the ensued pairwise comparisons were significant (all *P* values < 0.000001). Regarding the vertical growth patterns, only the ANOVA comparisons pertaining to the FMA and the lower facial height had become significantly different among the vertical growth patterns (Table 2). The Tamhane post hoc pairwise comparisons were all significant (all *P* values < 0.000001).

### MF position

**Fig. 4:**
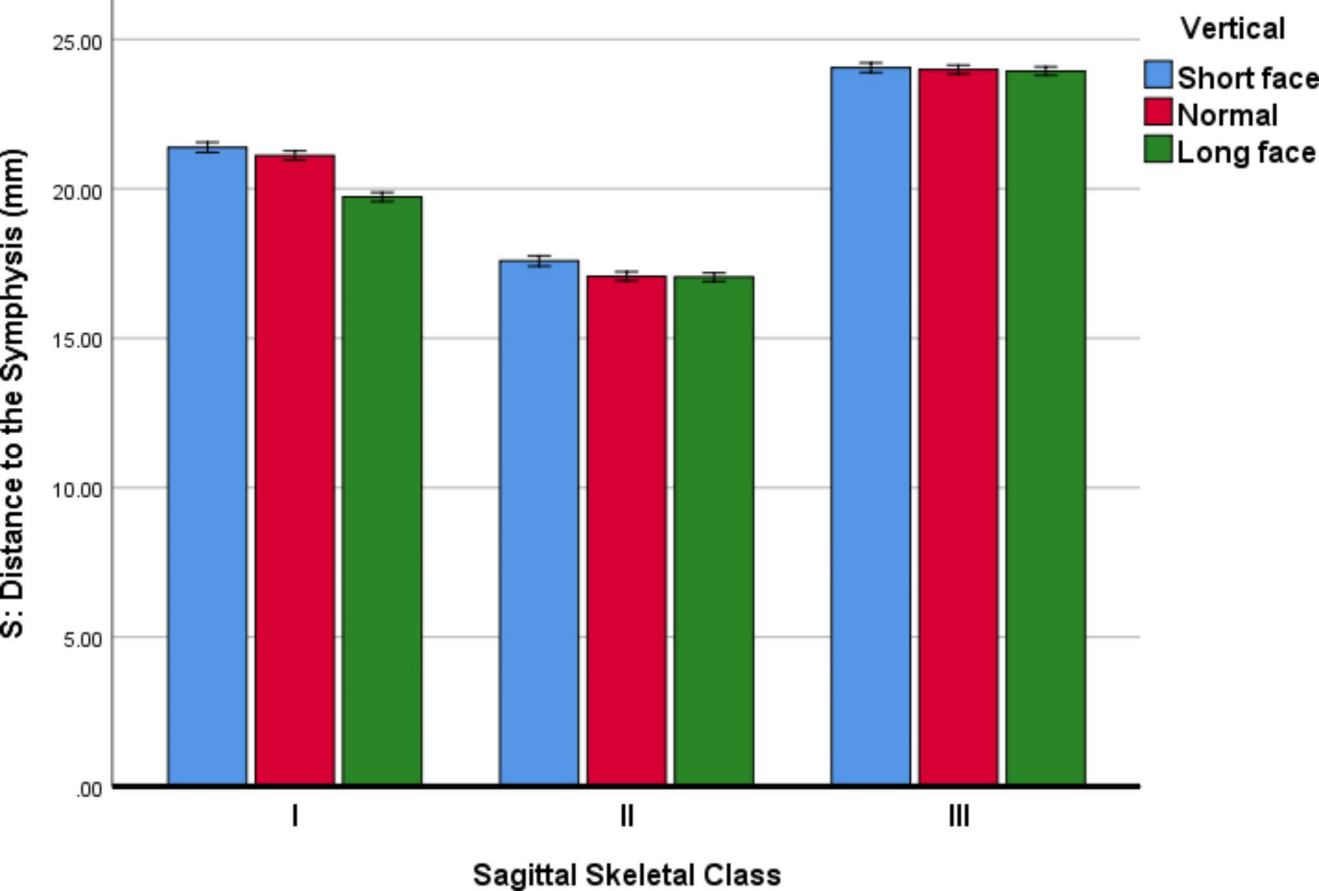
Means (and 95% CI) for the variable S (in mm, the perpendicular distance to the symphysis on the axial plane) in different Classes and vertical growth patterns. This distance shows the horizontal “width” dimension

**Fig. 5:**
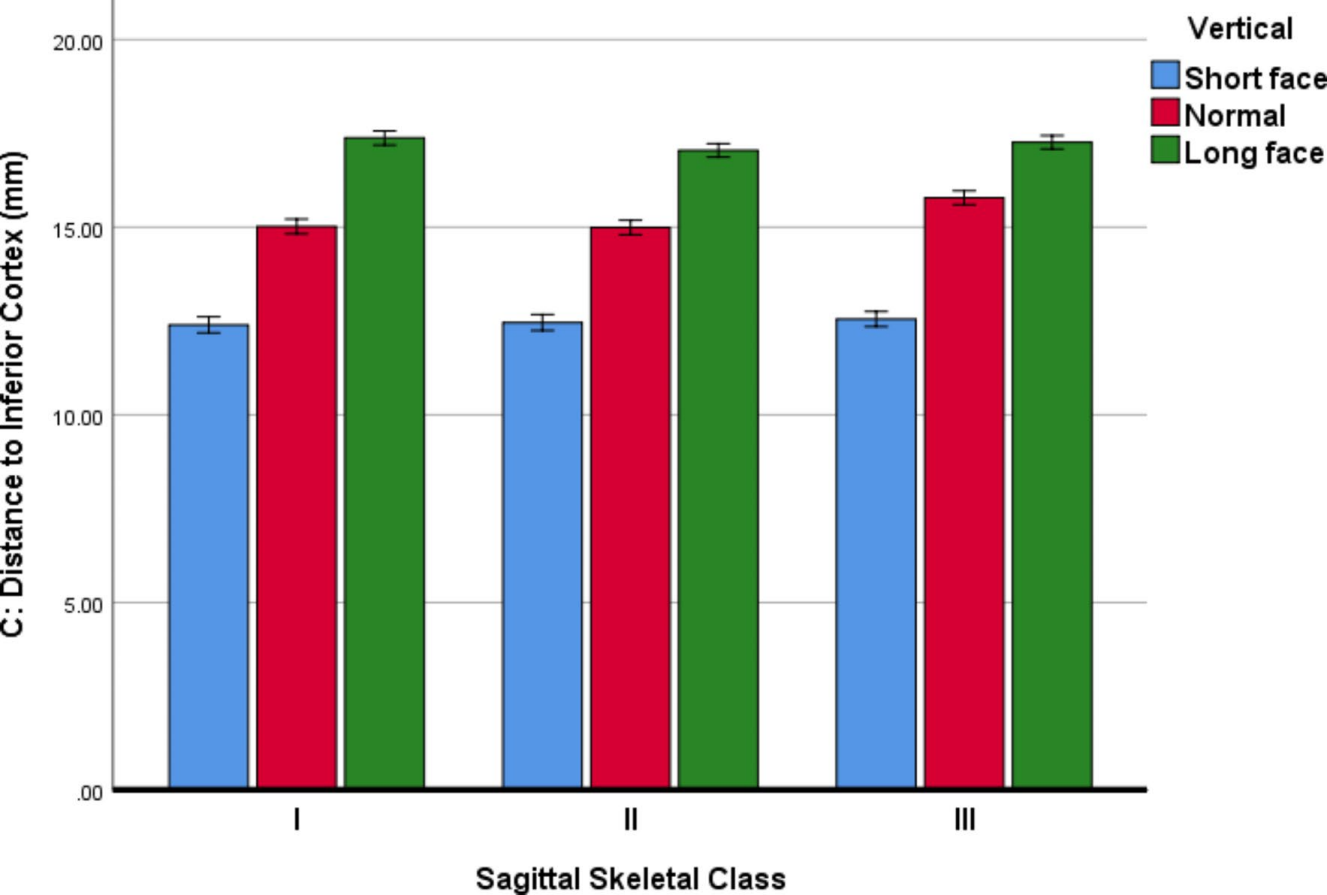
Means (and 95% CI) for the variable C (in mm, the perpendicular distance to the inferior cortex of the mandible on the cross-sectional plane) in different Classes and vertical growth patterns. This distance represents the inferior-superior “height” dimension

**Fig. 6:**
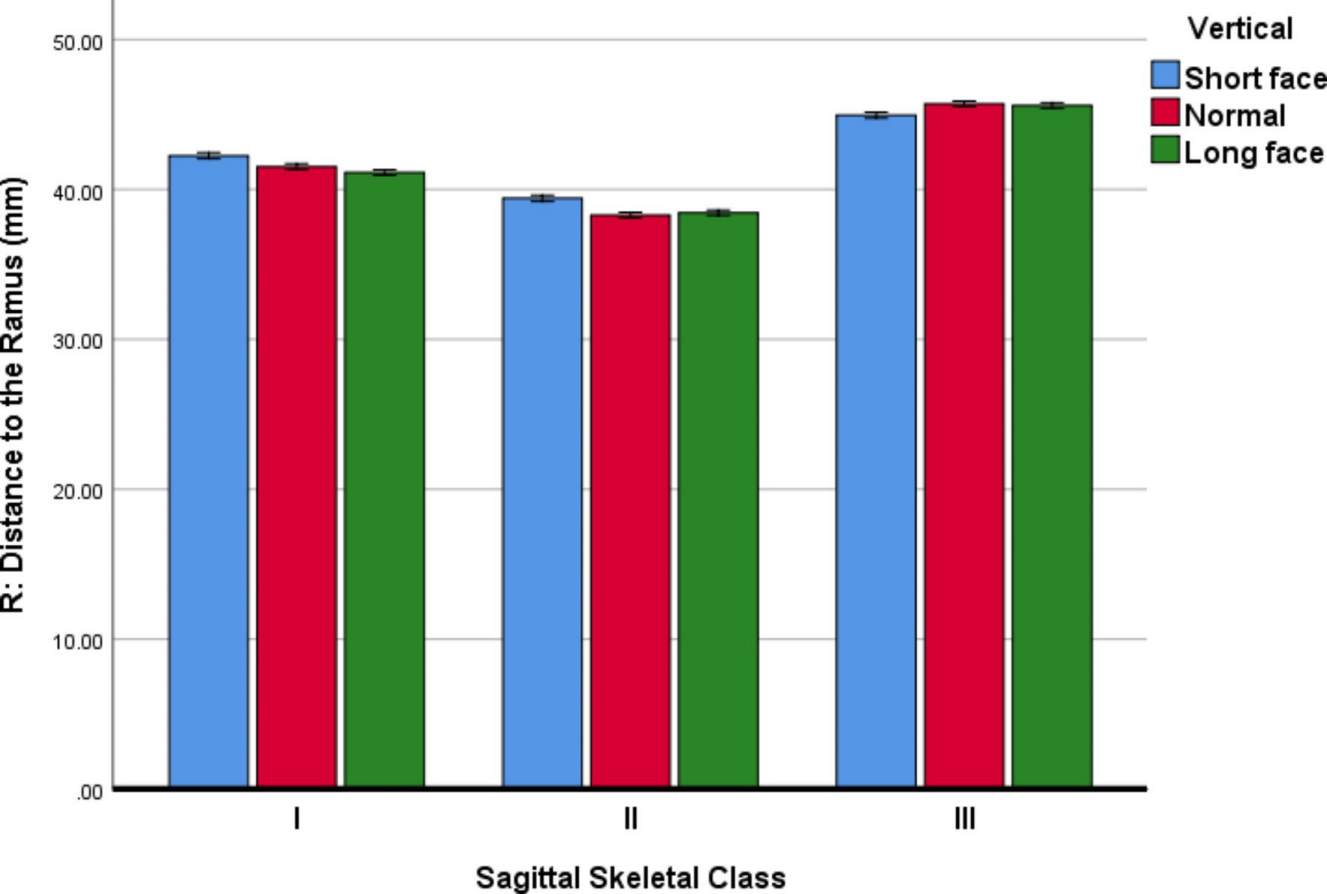
Means (and 95% CI) for the variable R (in mm, the perpendicular distance to the anterior border of the ramus on the parasagittal plane) in different Classes and vertical growth patterns. This distance indicates the anterior-posterior “length” dimension

Descriptive statistics and 95% CIs for the MF parameters in all the sub-groups are presented in Table 3; Figs. 4, 5 and 6.

- *S (Width): Perpendicular distance to the symphysis in the axial plane*.

For the parameter S (width), the 3-way ANCOVA (adjusted R-squared = 0.974, Fig. 4) showed that the effects of age (*P* = 0.078) and sex (*P* = 0.170) were insignificant. The effects of horizontal skeletal Classes (*P* < 0.000001) and vertical growth patterns (*P* < 0.000001) were significant. Regarding skeletal Classes, all pairwise comparisons became significant (all *P* values < 0.000001, Bonferroni); S was the smallest in Class II and longest in Class III cases. Similarly, all pairwise comparisons between different vertical growth patterns became significant (all *P* values ≤ 0.00008); S was the shortest in long faces and the largest in short faces. The only significant interaction was that of horizontal skeletal Classes and vertical growth patterns (*P* < 0.000001).

- *C (Height): Perpendicular distance to the mandibular inferior cortex on the cross-sectional plane*.

For the parameter C (height), the 3-way ANCOVA (adjusted R-squared = 0.922, Fig. 5) showed that the effects of age (*P* = 0.198) and sex (*P* = 0.886) were non-significant. The effects of horizontal skeletal Classes (*P* = 0.00002) and vertical growth patterns (*P* < 0.000001) were significant. Regarding skeletal Classes, pairwise comparisons between Class III and each of Classes I or II became significant (both *P* values ≤ 0.003, Bonferroni), C being larger in Class III than both Classes I and II. However, there was not a significant difference between Classes I and II in terms of the parameter C (height) (*P* = 0.684). All pairwise comparisons between different vertical growth patterns became significant (all *P* values < 0.000001); C was the largest in long faces and smallest in short faces. The only significant interaction was between horizontal skeletal Classes and vertical growth patterns (*P* = 0.00002).

- *R (Length): Perpendicular distance to the anterior border of the ramus on the parasagittal plane*.

For the parameter R (length), the 3-way ANCOVA (adjusted R-squared = 0.960, Fig. 6) showed that the effects of age (*P* = 0.065) and sex (*P* = 0.979) were insignificant. The effects of horizontal skeletal Classes (*P* < 0.000001) and vertical growth patterns (*P* < 0.000001) were significant. Regarding skeletal Classes, all pairwise comparisons became significant (all *P* values < 0.000001, Bonferroni); R was the largest in Class III and smallest in Class II. In terms of vertical growth patterns, pairwise comparisons between R values in short-face people versus normal- or long-face individuals were significant (both *P* values ≤ 0.00003, Bonferroni) with short-face patients having the largest R values. However, there was not a significant difference between R values measured in long faces versus normal faces (*P* = 0.448). The only significant interaction was that of horizontal skeletal Classes and vertical growth patterns (*P* < 0.000001).

**Table 1:** Descriptive statistics and 95% CIs for cephalometric variables in different skeletal Classes. The *P* values are computed using the one-way ANOVA.

Parameter	Class	N	Mean	SD	95% CI		Min	Max	*P*
**SNA**	**I**	120	80.55	0.73	80.42	80.68	79.0	82.0	**< 0.000001**
	**II**	120	82.88	1.39	82.63	83.13	80.0	85.0	
	**III**	120	79.63	1.09	79.44	79.83	78.0	82.0	
	**Total**	360	81.02	1.76	80.84	81.20	78.0	85.0	
**SNB**	**I**	120	79.23	0.78	79.08	79.37	78.0	81.0	**< 0.000001**
	**II**	120	77.31	1.14	77.10	77.51	76.0	79.0	
	**III**	120	83.08	1.54	82.81	83.36	81.0	88.0	
	**Total**	360	79.87	2.68	79.59	80.15	76.0	88.0	
**ANB**	**I**	120	1.32	0.74	1.18	1.45	0.0	2.0	**< 0.000001**
	**II**	120	5.58	1.59	5.29	5.86	2.0	9.0	
	**III**	120	-3.45	1.60	-3.74	-3.16	-7.0	-1.0	
	**Total**	360	1.15	3.94	0.74	1.56	-7.0	9.0	
**Facial Angle**	**I**	120	89.89	1.42	89.63	90.15	87.0	92.9	**< 0.000001**
	**II**	120	84.37	1.25	84.14	84.59	81.5	88.3	
	**III**	120	94.50	0.82	94.36	94.65	92.6	95.9	
	**Total**	360	89.59	4.32	89.14	90.03	81.5	95.9	
**FMA**	**I**	120	26.04	6.71	24.82	27.25	16.1	35.9	0.992
	**II**	120	26.11	6.55	24.92	27.29	16.1	35.9	
	**III**	120	26.15	6.64	24.94	27.35	16.1	35.9	
	**Total**	360	26.10	6.62	25.41	26.78	16.1	35.9	
**Lower Facial Height**	**I**	120	45.32	3.45	44.70	45.94	39.2	50.8	0.854
	**II**	120	45.07	3.68	44.40	45.74	39.1	50.8	
	**III**	120	45.13	3.61	44.48	45.78	39.0	50.9	
	**Total**	360	45.17	3.57	44.80	45.54	39.0	50.9	

**SD**, standard deviation; **Min**, minimum; **Max**, maximum

**Table 2:** Descriptive statistics and 95% CIs for cephalometric variables in different vertical growth patterns. The *P* values are computed using the one-way ANOVA.

Parameter	Vertical growth	N	Mean	SD	95% CI		Min	Max	*P*
**SNA**	**Short face**	120	81.09	1.91	80.75	81.44	78.0	85.0	0.855
	**Normal face**	120	80.97	1.69	80.66	81.27	78.0	85.0	
	**Long face**	120	81.01	1.68	80.71	81.31	78.0	85.0	
	**Total**	360	81.02	1.76	80.84	81.20	78.0	85.0	
**SNB**	**Short face**	120	79.86	2.74	79.36	80.35	76.0	88.0	0.899
	**Normal face**	120	79.80	2.60	79.33	80.27	76.0	85.0	
	**Long face**	120	79.96	2.73	79.47	80.45	76.0	85.0	
	**Total**	360	79.87	2.68	79.59	80.15	76.0	88.0	
**ANB**	**Short face**	120	1.23	4.08	0.50	1.97	-7.0	9.0	0.937
	**Normal face**	120	1.16	3.84	0.46	1.85	-6.0	9.0	
	**Long face**	120	1.05	3.92	0.34	1.76	-6.0	9.0	
	**Total**	360	1.15	3.94	0.74	1.56	-7.0	9.0	
**Facial Angle**	**Short face**	120	89.70	4.23	88.93	90.46	82.1	95.9	0.708
	**Normal face**	120	89.32	4.52	88.50	90.14	81.5	95.8	
	**Long face**	120	89.74	4.22	88.98	90.51	82.1	95.8	
	**Total**	360	89.59	4.32	89.14	90.03	81.5	95.9	
**FMA**	**Short face**	120	18.80	1.64	18.50	19.09	16.1	21.9	**< 0.000001**
	**Normal face**	120	25.45	3.16	24.88	26.02	20.0	30.7	
	**Long face**	120	34.05	1.23	33.82	34.27	32.0	35.9	
	**Total**	360	26.10	6.62	25.41	26.78	16.1	35.9	
**Lower Facial Height**	**Short face**	120	41.04	1.13	40.83	41.24	39.0	43.0	**< 0.000001**
	**Normal face**	120	44.98	0.59	44.87	45.09	44.0	46.0	
	**Long face**	120	49.50	0.85	49.35	49.65	48.0	50.9	
	**Total**	360	45.17	3.57	44.80	45.54	39.0	50.9	

**SD**, standard deviation; **Min**, minimum; **Max**, maximum

**Table 3:** The MF parameters in all subgroups (in mm)

Class	Vertical	Mand Plane	MF	Sex	N	Mean	SD	95% CI		Min	Max
**I**	**Short face**	**Low**	**S (width)**	**Female**	22	21.36	0.51	21.13	21.59	20.5	22.1
				**Male**	7	21.41	0.46	20.99	21.84	20.8	21.9
			**C (height)**	**Female**	22	12.34	0.37	12.17	12.50	11.7	13.1
				**Male**	7	12.39	0.29	12.12	12.65	11.9	12.8
			**R (length)**	**Female**	22	42.47	0.65	42.18	42.76	40.8	43.9
				**Male**	7	42.13	0.48	41.68	42.57	41.7	43.0
		**Normal**	**S (width)**	**Female**	9	21.42	0.49	21.05	21.80	20.7	22.0
				**Male**	2	21.40	0.42	17.59	25.21	21.1	21.7
			**C (height)**	**Female**	9	12.43	0.45	12.09	12.78	11.8	13.1
				**Male**	2	12.65	0.64	6.93	18.37	12.2	13.1
			**R (length)**	**Female**	9	42.03	1.00	41.26	42.80	40.0	43.8
				**Male**	2	42.35	0.78	35.36	49.34	41.8	42.9
	**Normal**	**Low**	**S (width)**	**Female**	2	21.40	0.00	21.40	21.40	21.4	21.4
				**Male**	2	20.80	0.14	19.53	22.07	20.7	20.9
			**C (height)**	**Female**	2	14.65	0.49	10.20	19.10	14.3	15.0
				**Male**	2	15.10	0.57	10.02	20.18	14.7	15.5
			**R (length)**	**Female**	2	41.60	0.57	36.52	46.68	41.2	42.0
				**Male**	2	41.60	0.42	37.79	45.41	41.3	41.9
		**Normal**	**S (width)**	**Female**	19	21.07	0.42	20.87	21.28	20.0	21.8
				**Male**	7	21.07	0.31	20.79	21.36	20.6	21.4
			**C (height)**	**Female**	19	14.96	0.63	14.66	15.26	14.1	16.7
				**Male**	7	15.13	0.80	14.39	15.87	14.4	16.8
			**R (length)**	**Female**	19	41.58	0.53	41.32	41.84	40.8	42.5
				**Male**	7	41.43	0.52	40.95	41.91	40.9	42.0
		**High**	**S (width)**	**Female**	7	20.97	0.34	20.66	21.29	20.4	21.5
				**Male**	3	21.63	0.21	21.12	22.15	21.4	21.8
			**C (height)**	**Female**	7	14.84	0.65	14.24	15.44	14.0	15.7
				**Male**	3	15.20	0.60	13.71	16.69	14.6	15.8
			**R (length)**	**Female**	7	41.66	0.47	41.22	42.09	41.1	42.4
				**Male**	3	41.40	0.87	39.25	43.55	40.9	42.4
	**Long face**	**Normal**	**S (width)**	**Female**	7	19.99	0.93	19.13	20.84	18.9	21.4
				**Male**	8	19.85	0.93	19.07	20.63	18.7	21.2
			**C (height)**	**Female**	7	17.44	0.20	17.26	17.63	17.1	17.7
				**Male**	8	17.31	0.30	17.06	17.56	16.9	17.7
			**R (length)**	**Female**	7	41.20	0.64	40.61	41.79	40.6	42.4
				**Male**	8	41.30	0.41	40.95	41.65	40.6	42.0
		**High**	**S (width)**	**Female**	19	19.55	0.68	19.22	19.87	18.4	20.8
				**Male**	6	19.67	0.55	19.09	20.24	18.7	20.2
			**C (height)**	**Female**	19	17.38	0.43	17.17	17.58	16.8	18.1
				**Male**	6	17.38	0.50	16.86	17.91	16.8	18.2
			**R (length)**	**Female**	19	41.06	0.63	40.75	41.36	40.1	42.8
				**Male**	6	40.93	0.61	40.30	41.57	40.2	41.7
**II**	**Short face**	**Low**	**S (width)**	**Female**	22	17.44	0.40	17.26	17.62	16.7	18.2
				**Male**	9	17.71	0.39	17.41	18.01	17.0	18.2
			**C (height)**	**Female**	22	12.37	0.47	12.16	12.58	11.6	13.3
				**Male**	9	12.49	0.37	12.20	12.78	11.9	13.1
			**R (length)**	**Female**	22	39.18	0.66	38.89	39.47	37.5	40.3
				**Male**	9	39.43	0.49	39.05	39.81	38.7	40.2
	**Normal**	**Low**	**S (width)**	**Female**	6	17.00	0.28	16.70	17.30	16.7	17.5
				**Male**	3	17.17	0.06	17.02	17.31	17.1	17.2
			**C (height)**	**Female**	6	15.27	0.16	15.10	15.44	15.0	15.5
				**Male**	3	15.20	0.10	14.95	15.45	15.1	15.3
			**R (length)**	**Female**	6	38.23	0.53	37.68	38.79	37.7	39.0
				**Male**	3	37.97	0.12	37.68	38.25	37.9	38.1
		**Normal**	**S (width)**	**Female**	14	17.09	0.26	16.93	17.24	16.8	17.7
				**Male**	8	17.04	0.27	16.81	17.27	16.6	17.5
			**C (height)**	**Female**	14	15.02	0.29	14.85	15.19	14.6	15.6
				**Male**	8	14.88	0.31	14.62	15.13	14.3	15.1
			**R (length)**	**Female**	14	38.16	0.38	37.94	38.38	37.7	39.1
				**Male**	8	38.46	0.48	38.06	38.86	37.8	38.9
		**High**	**S (width)**	**Female**	7	17.16	0.32	16.86	17.45	16.9	17.6
				**Male**	2	16.95	0.07	16.31	17.59	16.9	17.0
			**C (height)**	**Female**	7	14.86	0.27	14.61	15.11	14.4	15.2
				**Male**	2	14.85	0.07	14.21	15.49	14.8	14.9
			**R (length)**	**Female**	7	38.37	0.51	37.90	38.84	37.8	39.1
				**Male**	2	38.30	0.42	34.49	42.11	38.0	38.6
	**Long face**	**Normal**	**S (width)**	**Female**	11	16.69	0.39	16.43	16.95	16.1	17.3
				**Male**	5	17.38	0.45	16.82	17.94	16.9	18.0
			**C (height)**	**Female**	11	16.69	0.39	16.43	16.95	16.1	17.3
				**Male**	5	17.38	0.45	16.82	17.94	16.9	18.0
			**R (length)**	**Female**	11	38.43	0.97	37.77	39.08	37.5	40.2
				**Male**	5	38.36	1.06	37.04	39.68	37.6	40.2
		**High**	**S (width)**	**Female**	12	17.13	0.58	16.75	17.50	16.3	18.3
				**Male**	12	17.09	0.37	16.86	17.33	16.6	17.9
			**C (height)**	**Female**	12	17.13	0.58	16.75	17.50	16.3	18.3
				**Male**	12	17.09	0.37	16.86	17.33	16.6	17.9
			**R (length)**	**Female**	12	38.43	0.80	37.92	38.94	37.0	39.9
				**Male**	12	38.49	0.81	37.98	39.01	37.6	40.7
**III**	**Short face**	**Low**	**S (width)**	**Female**	14	23.95	0.41	23.71	24.19	23.1	24.5
				**Male**	5	24.10	0.19	23.87	24.33	23.8	24.3
			**C (height)**	**Female**	14	12.51	0.52	12.21	12.81	11.8	13.5
				**Male**	5	12.52	0.55	11.83	13.21	11.8	13.2
			**R (length)**	**Female**	14	44.96	0.30	44.79	45.14	44.6	45.7
				**Male**	5	45.04	0.38	44.56	45.52	44.6	45.5
		**Normal**	**S (width)**	**Female**	15	24.05	0.37	23.85	24.26	23.5	24.6
				**Male**	6	24.08	0.34	23.72	24.44	23.7	24.6
			**C (height)**	**Female**	15	12.77	0.47	12.51	13.03	12.0	13.9
				**Male**	6	12.40	0.18	12.21	12.59	12.1	12.6
			**R (length)**	**Female**	15	44.90	0.33	44.72	45.08	44.3	45.6
				**Male**	6	44.88	0.13	44.74	45.02	44.7	45.1
	**Normal**	**Normal**	**S (width)**	**Female**	9	24.04	0.19	23.90	24.19	23.8	24.4
				**Male**	3	23.93	0.23	23.36	24.51	23.8	24.2
			**C (height)**	**Female**	9	16.04	0.96	15.30	16.78	14.7	17.0
				**Male**	3	16.53	1.27	13.39	19.68	15.1	17.5
			**R (length)**	**Female**	9	45.70	0.31	45.46	45.94	45.2	46.1
				**Male**	3	45.73	0.21	45.22	46.25	45.5	45.9
		**High**	**S (width)**	**Female**	16	24.04	0.22	23.93	24.16	23.7	24.5
				**Male**	12	23.94	0.17	23.84	24.05	23.7	24.3
			**C (height)**	**Female**	16	15.68	1.21	15.04	16.32	14.5	17.6
				**Male**	12	15.57	1.15	14.83	16.30	14.1	17.5
			**R (length)**	**Female**	16	45.60	0.39	45.39	45.81	45.0	46.1
				**Male**	12	45.78	0.33	45.58	45.99	45.2	46.1
	**Long face**	**Normal**	**S (width)**	**Female**	8	23.85	0.63	23.33	24.37	23.2	25.1
				**Male**	5	24.12	0.67	23.29	24.95	23.7	25.3
			**C (height)**	**Female**	8	17.46	0.48	17.06	17.87	16.8	18.1
				**Male**	5	17.04	0.36	16.59	17.49	16.6	17.5
			**R (length)**	**Female**	8	45.79	0.58	45.30	46.27	44.9	46.6
				**Male**	5	45.64	0.43	45.11	46.17	44.9	46.0
		**High**	**S (width)**	**Female**	15	24.02	0.46	23.77	24.27	23.0	24.8
				**Male**	12	23.83	0.59	23.46	24.21	22.9	25.0
			**C (height)**	**Female**	15	17.29	0.45	17.04	17.53	16.6	18.0
				**Male**	12	17.24	0.48	16.93	17.55	16.6	18.2
			**R (length)**	**Female**	15	45.55	0.41	45.33	45.78	44.9	46.1
				**Male**	12	45.53	0.38	45.29	45.77	45.0	46.1

**Mand**, mandibular; **MF**, mental foramen; **SD**, standard deviation; **Min**, minimum; **Max**, maximum. **S**, Perpendicular distance to the symphysis on the axial plane; **C**, Perpendicular distance to the inferior cortex of the mandible on the cross-sectional plane; **R**, Perpendicular distance to the anterior border of the ramus on the parasagittal plane

**Table 4:** The position of mental foramen (in mm) in different skeletal Classes and vertical growth patterns. The *P* values are calculated using the one-way ANOVA.

Parameter	Type	N	Mean	SD	95% CI		Min	Max	*P*
**S (width)**	**I**	120	20.73	0.93	20.56	20.90	18.4	22.1	**< 0.000001**
	**II**	120	17.20	0.45	17.12	17.28	16.1	18.3	
	**III**	120	23.99	0.39	23.92	24.06	22.9	25.3	
	**Total**	360	20.64	2.85	20.35	20.94	16.1	25.3	
**C (height)**	**I**	120	14.91	2.10	14.53	15.29	11.7	18.2	0.289
	**II**	120	14.82	1.93	14.47	15.17	11.6	18.3	
	**III**	120	15.22	2.10	14.84	15.60	11.8	18.2	
	**Total**	360	14.98	2.05	14.77	15.20	11.6	18.3	
**R (length)**	**I**	120	41.66	0.78	41.52	41.80	40.0	43.9	**< 0.000001**
	**II**	120	38.69	0.83	38.54	38.84	37.0	40.8	
	**III**	120	45.41	0.49	45.32	45.50	44.3	46.6	
	**Total**	360	41.92	2.84	41.63	42.21	37.0	46.6	
**S (width)**	**Short face**	120	20.97	2.72	20.48	21.46	16.7	24.6	0.116
	**Normal face**	120	20.73	2.87	20.21	21.25	16.6	24.5	
	**Long face**	120	20.22	2.92	19.70	20.75	16.1	25.3	
	**Total**	360	20.64	2.85	20.35	20.94	16.1	25.3	
**C (height)**	**Short face**	120	12.47	0.44	12.39	12.55	11.6	13.9	**< 0.000001**
	**Normal face**	120	15.26	0.85	15.10	15.41	14.0	17.6	
	**Long face**	120	17.23	0.47	17.14	17.31	16.1	18.3	
	**Total**	360	14.98	2.05	14.77	15.20	11.6	18.3	
**R (length)**	**Short face**	120	42.21	2.35	41.78	42.63	37.5	45.7	0.381
	**Normal face**	120	41.83	3.08	41.28	42.39	37.7	46.1	
	**Long face**	120	41.72	3.04	41.17	42.27	37.0	46.6	
	**Total**	360	41.92	2.84	41.63	42.21	37.0	46.6	

**SD**, standard deviation; **Min**, minimum; **Max**, maximum. **S**, Perpendicular distance to the symphysis on the axial plane; **C**, Perpendicular distance to the inferior cortex of the mandible on the cross-sectional plane; **R**, Perpendicular distance to the anterior border of the ramus on the parasagittal plane

### Correlations between age with the 3 MF parameters

The Pearson coefficient showed no significant correlations between age with each of the 3 MF parameters (each n = 360, coefficients ranged between − 0.015 and − 0.029, all 3 *P* values ≥ 0.579).

### Differences in the MF position across horizontal classes I, II, and III

- *In the whole sample*.

According to the one-way ANOVA, there were significant differences among skeletal Classes I to III in the case of the MF parameters S and R (Table 4). All the Tamhane post hoc comparisons were significant (all *P* values < 0.000001).

- *In males and females, separately*.

In each of the sexes, both parameters S and R were significantly different across Classes I, II, and III (Table 5). All Tamhane pairwise comparisons were significant (all *P* values < 0.000001). However, in either of the sexes assessed separately, the parameter C (height) was not different among the Classes (Table 5).

- *Within different vertical growth patterns*.

The only insignificant ANOVA comparison (*P* = 0.092) was for the parameter C (height) compared among the Classes within the ‘short-face’ group (Table 6). Except for this ANOVA comparison, all other ANOVA comparisons performed among Classes I, II, and III separately in each of the groups ‘short, long, and normal face’ were significant (Table 6). The Tamhane pairwise comparisons of the parameters S and R became all significant (all *P* values < 0.000001). In the case of the parameter C (height) in normal-face patients, the Tamhane test following the significant ANOVAs showed that the pairwise comparison of the parameter C (height) between Classes I and II were not significant (*P* = 0.992), but the other two were significant (both *P* values < 0.001). In the case of the parameter C (height) in long-face patients, the only significant pairwise comparison was seen between Classes I and II (*P* = 0.002), and the other two were insignificant (*P* ≥ 0.06).

**Table 5:** The position of mental foramen (in mm) in different skeletal Classes and vertical growth patterns, separately in males and females. The *P* values are calculated using the one-way ANOVA.

Sex	Parameter	Type	N	Mean	SD	95% CI		Min	Max	*P*
**Female**	**S (width)**	**I**	85	20.75	0.92	20.55	20.95	18.4	22.1	**< 0.000001**
		**II**	81	17.18	0.46	17.07	17.28	16.1	18.3	
		**III**	77	24.00	0.38	23.92	24.09	23.0	25.1	
		**Total**	243	20.59	2.83	20.23	20.95	16.1	25.1	
	**C (height)**	**I**	85	14.74	2.12	14.28	15.20	11.7	18.1	0.289
		**II**	81	14.57	1.91	14.14	14.99	11.6	18.3	
		**III**	77	15.08	2.13	14.59	15.56	11.8	18.1	
		**Total**	243	14.79	2.06	14.53	15.05	11.6	18.3	
	**R (length)**	**I**	85	41.72	0.82	41.54	41.89	40.0	43.9	**< 0.000001**
		**II**	81	38.72	0.85	38.53	38.91	37.0	40.8	
		**III**	77	45.37	0.51	45.25	45.49	44.3	46.6	
		**Total**	243	41.88	2.79	41.52	42.23	37.0	46.6	
**Male**	**S (width)**	**I**	35	20.67	0.94	20.35	20.99	18.7	21.9	**< 0.000001**
		**II**	39	17.26	0.43	17.12	17.40	16.6	18.2	
		**III**	43	23.97	0.42	23.84	24.10	22.9	25.3	
		**Total**	117	20.75	2.89	20.22	21.27	16.6	25.3	
	**C (height)**	**I**	35	15.33	2.03	14.63	16.03	11.9	18.2	0.937
		**II**	39	15.35	1.90	14.74	15.97	11.9	18.0	
		**III**	43	15.48	2.05	14.85	16.11	11.8	18.2	
		**Total**	117	15.39	1.98	15.03	15.75	11.8	18.2	
	**R (length)**	**I**	35	41.51	0.66	41.29	41.74	40.2	43.0	**< 0.000001**
		**II**	39	38.64	0.79	38.38	38.89	37.6	40.7	
		**III**	43	45.48	0.46	45.34	45.62	44.6	46.1	
		**Total**	117	42.01	2.96	41.47	42.56	37.6	46.1	
**Female**	**S (width)**	**Short face**	91	20.87	2.73	20.31	21.44	16.7	24.6	0.271
		**Normal**	80	20.66	2.85	20.02	21.29	16.7	24.5	
		**Long face**	72	20.16	2.92	19.47	20.85	16.1	25.1	
		**Total**	243	20.59	2.83	20.23	20.95	16.1	25.1	
	**C (height)**	**Short face**	91	12.47	0.47	12.37	12.57	11.6	13.9	**< 0.000001**
		**Normal**	80	15.23	0.83	15.05	15.42	14.0	17.6	
		**Long face**	72	17.23	0.50	17.11	17.35	16.1	18.3	
		**Total**	243	14.79	2.06	14.53	15.05	11.6	18.3	
	**R (length)**	**Short face**	91	42.15	2.36	41.66	42.64	37.5	45.7	0.489
		**Normal**	80	41.72	3.03	41.05	42.40	37.7	46.1	
		**Long face**	72	41.69	3.01	40.99	42.40	37.0	46.6	
		**Total**	243	41.88	2.79	41.52	42.23	37.0	46.6	
**Male**	**S (width)**	**Short face**	29	21.28	2.71	20.25	22.31	17.0	24.6	0.352
		**Normal**	40	20.87	2.93	19.93	21.81	16.6	24.3	
		**Long face**	48	20.32	2.95	19.46	21.18	16.6	25.3	
		**Total**	117	20.75	2.89	20.22	21.27	16.6	25.3	
	**C (height)**	**Short face**	29	12.46	0.36	12.33	12.60	11.8	13.2	**< 0.000001**
		**Normal**	40	15.31	0.89	15.02	15.60	14.1	17.5	
		**Long face**	48	17.23	0.41	17.11	17.35	16.6	18.2	
		**Total**	117	15.39	1.98	15.03	15.75	11.8	18.2	
	**R (length)**	**Short face**	29	42.38	2.36	41.48	43.28	38.7	45.5	0.670
		**Normal**	40	42.06	3.20	41.03	43.08	37.8	46.1	
		**Long face**	48	41.76	3.11	40.85	42.66	37.6	46.1	
		**Total**	117	42.01	2.96	41.47	42.56	37.6	46.1	

**SD**, standard deviation; **Min**, minimum; **Max**, maximum. **S**, Perpendicular distance to the symphysis on the axial plane; **C**, Perpendicular distance to the inferior cortex of the mandible on the cross-sectional plane; **R**, Perpendicular distance to the anterior border of the ramus on the parasagittal plane

**Table 6:** The position of mental foramen (in mm) in skeletal Classes in different vertical patterns. The *P* values are calculated using the one-way ANOVA.

Type	Parameter	Class	N	Mean	SD	95% CI		Min	Max	*P*
**Short face**	**S (width)**	**I**	40	21.39	0.48	21.23	21.54	20.5	22.1	**< 0.000001**
		**II**	40	17.51	0.40	17.38	17.63	16.7	18.2	
		**III**	40	24.03	0.36	23.91	24.14	23.1	24.6	
	**C (height)**	**I**	40	12.38	0.38	12.26	12.50	11.7	13.1	0.092
		**II**	40	12.43	0.46	12.29	12.58	11.6	13.3	
		**III**	40	12.59	0.47	12.44	12.74	11.8	13.9	
	**R (length)**	**I**	40	42.31	0.72	42.07	42.54	40.0	43.9	**< 0.000001**
		**II**	40	39.38	0.65	39.17	39.59	37.5	40.8	
		**III**	40	44.94	0.30	44.84	45.03	44.3	45.7	
**Normal**	**S (width)**	**I**	40	21.10	0.39	20.98	21.22	20.0	21.8	**< 0.000001**
		**II**	40	17.08	0.26	16.99	17.16	16.6	17.7	
		**III**	40	24.01	0.20	23.94	24.07	23.7	24.5	
	**C (height)**	**I**	40	14.98	0.63	14.78	15.18	14.0	16.8	**0.000002**
		**II**	40	15.01	0.29	14.91	15.10	14.3	15.6	
		**III**	40	15.79	1.13	15.43	16.16	14.1	17.6	
	**R (length)**	**I**	40	41.56	0.51	41.39	41.72	40.8	42.5	**< 0.000001**
		**II**	40	38.26	0.44	38.12	38.40	37.7	39.1	
		**III**	40	45.69	0.34	45.58	45.80	45.0	46.1	
**Long face**	**S (width)**	**I**	40	19.70	0.75	19.46	19.94	18.4	21.4	**< 0.000001**
		**II**	40	17.03	0.50	16.87	17.19	16.1	18.3	
		**III**	40	23.94	0.55	23.77	24.12	22.9	25.3	
	**C (height)**	**I**	40	17.38	0.37	17.26	17.50	16.8	18.2	**0.002**
		**II**	40	17.03	0.50	16.87	17.19	16.1	18.3	
		**III**	40	17.28	0.46	17.13	17.42	16.6	18.2	
	**R (length)**	**I**	40	41.11	0.58	40.93	41.30	40.1	42.8	**< 0.000001**
		**II**	40	38.44	0.85	38.17	38.71	37.0	40.7	
		**III**	40	45.61	0.44	45.47	45.74	44.9	46.6	

**SD**, standard deviation; **Min**, minimum; **Max**, maximum. **S**, Perpendicular distance to the symphysis on the axial plane; **C**, Perpendicular distance to the inferior cortex of the mandible on the cross-sectional plane; **R**, Perpendicular distance to the anterior border of the ramus on the parasagittal plane

**Table 7:** The position of mental foramen (in mm) in vertical patterns within different skeletal Classes. The *P* values are calculated using the one-way ANOVA.

Class	Parameter	Type	N	Mean	SD	95% CI		Min	Max	*P*
**I**	**S (width)**	**Short face**	40	21.39	0.48	21.23	21.54	20.5	22.1	**< 0.000001**
		**Normal**	40	21.10	0.39	20.98	21.22	20.0	21.8	
		**Long face**	40	19.70	0.75	19.46	19.94	18.4	21.4	
	**C (height)**	**Short face**	40	12.38	0.38	12.26	12.50	11.7	13.1	**< 0.000001**
		**Normal**	40	14.98	0.63	14.78	15.18	14.0	16.8	
		**Long face**	40	17.38	0.37	17.26	17.50	16.8	18.2	
	**R (length)**	**Short face**	40	42.31	0.72	42.07	42.54	40.0	43.9	**< 0.000001**
		**Normal**	40	41.56	0.51	41.39	41.72	40.8	42.5	
		**Long face**	40	41.11	0.58	40.93	41.30	40.1	42.8	
**II**	**S (width)**	**Short face**	40	17.51	0.40	17.38	17.63	16.7	18.2	**< 0.000001**
		**Normal**	40	17.08	0.26	16.99	17.16	16.6	17.7	
		**Long face**	40	17.03	0.50	16.87	17.19	16.1	18.3	
	**C (height)**	**Short face**	40	12.43	0.46	12.29	12.58	11.6	13.3	**< 0.000001**
		**Normal**	40	15.01	0.29	14.91	15.10	14.3	15.6	
		**Long face**	40	17.03	0.50	16.87	17.19	16.1	18.3	
	**R (length)**	**Short face**	40	39.38	0.65	39.17	39.59	37.5	40.8	**< 0.000001**
		**Normal**	40	38.26	0.44	38.12	38.40	37.7	39.1	
		**Long face**	40	38.44	0.85	38.17	38.71	37.0	40.7	
**III**	**S (width)**	**Short face**	40	24.03	0.36	23.91	24.14	23.1	24.6	0.609
		**Normal**	40	24.01	0.20	23.94	24.07	23.7	24.5	
		**Long face**	40	23.94	0.55	23.77	24.12	22.9	25.3	
	**C (height)**	**Short face**	40	12.59	0.47	12.44	12.74	11.8	13.9	**< 0.000001**
		**Normal**	40	15.79	1.13	15.43	16.16	14.1	17.6	
		**Long face**	40	17.28	0.46	17.13	17.42	16.6	18.2	
	**R (length)**	**Short face**	40	44.94	0.30	44.84	45.03	44.3	45.7	**< 0.000001**
		**Normal**	40	45.69	0.34	45.58	45.80	45.0	46.1	
		**Long face**	40	45.61	0.44	45.47	45.74	44.9	46.6	

**SD**, standard deviation; **Min**, minimum; **Max**, maximum. **S**, Perpendicular distance to the symphysis on the axial plane; **C**, Perpendicular distance to the inferior cortex of the mandible on the cross-sectional plane; **R**, Perpendicular distance to the anterior border of the ramus on the parasagittal plane

### Differences in the MF position across vertical growth patterns

- *In the whole sample*.

There was a significant difference among the vertical growth patterns only in the case of the MF parameter C (height) (Table 4). All the post hoc comparisons were significant (all *P* values < 0.000001).

- *In males and females separately*.

In each of the sexes, only the variable C was different across the ‘long, short, and normal’ vertical growth patterns (Table 5). All Tamhane pairwise comparisons were significant (all *P* values < 0.000001). However, in both sexes, the parameters S and R were not different among vertical growth patterns (Table 5).

- *Within each of Classes I, II, and III separately*.

The only ANOVA comparison that became insignificant was that of the parameter S (width) compared among vertical growth patterns within Class III patients (Table 7). All other ANOVA comparisons became significant (Table 7). Most of the Tamhane post hoc comparisons became significant (all significant *P* values ≤ 0.014). A few pairwise comparisons became insignificant: The parameter S (width) compared between long and normal face patterns within the Class II group (*P* = 0.933); the parameter R (length) compared between long and normal faces within the Class II group (*P* = 0.560); and the parameter R (length) compared between long and normal faces within the Class III group (*P* = 0.724).

### Differences between MF parameters in men versus women

The independent-samples t-test showed that only the parameter C (height) was significantly greater in males than in females (Table 8). The other two parameters did not show sex dimorphism (Table 8).

**Table 8:** Sex dimorphism in mental foramen parameters (in mm). The *P* values are calculated using the independent-samples t-test

Parameter	Sex	N	Mean	SD	95% CI		Min	Max	*P*
**S (width)**	**Female**	243	20.59	2.83	20.23	20.95	16.1	25.1	0.628
	**Male**	117	20.75	2.89	20.22	21.27	16.6	25.3	
	**Total**	360	20.64	2.85	20.35	20.94	16.1	25.3	
** C (height)**	**Female**	243	14.79	2.06	14.53	15.05	11.6	18.3	**0.009**
	**Male**	117	15.39	1.98	15.03	15.75	11.8	18.2	
	**Total**	360	14.98	2.05	14.77	15.20	11.6	18.3	
**R (length)**	**Female**	243	41.88	2.79	41.52	42.23	37.0	46.6	0.669
	**Male**	117	42.01	2.96	41.47	42.56	37.6	46.1	
	**Total**	360	41.92	2.84	41.63	42.21	37.0	46.6	

**SD**, standard deviation; **Min**, minimum; **Max**, maximum. **S**, Perpendicular distance to the symphysis on the axial plane; **C**, Perpendicular distance to the inferior cortex of the mandible on the cross-sectional plane; **R**, Perpendicular distance to the anterior border of the ramus on the parasagittal plane

## Discussion

The inferior alveolar nerve (IAN) injury is one of the most critical concerns during intraoral surgical processes, with a high prevalence of failure in dental anaesthetic techniques [25, 26]. The IAN injury may be a reversible event or can last for more than six months; it primarily occurs during implant placement, alveolar bone splitting procedures, and third molar extractions. Since the MF position varies in different ethnicities and age groups, it should be a critical risk factor in the occurrence of this complication [25]. In this regard, this study evaluated the position of the MF in different sagittal and vertical growth patterns using CBCT and found numerous differences in its position among different Classes or different vertical growth patterns. In this study, we observed that the variable S or “width” was the smallest in Class II and longest in Class III cases. The changes observed in this variable between the 3 vertical growth patterns were smaller than those in different Classes, but still significant: this distance was the shortest in long faces and the largest in short faces. The variable C or “height” was slightly but significantly larger in Class III cases than either of Class I or II patients; however, between Classes I and II there might not be a significant difference. This distance was the largest in long faces and smallest in short faces. The variable R or “length” was the largest in Class III and smallest in Class II. This variable was slightly larger in short-face people than in normal-face or long-face individuals; the latter two might not differ in terms of this distance. A recent 2022 study reported an effect of the skeletal Class and facial type on the MF dimensions in women [1]. Their results were less diverse than what was observed in this study. This might be due to their smaller sample as well as the use of different statistical approaches by them. Furthermore, Zmyslowska-Polakowska et al. (2019) [8] asserted that horizontal and vertical diameters were not divergent in different ages. However, those measures were significantly greater on the right side in males. Meanwhile, the MF types were not significantly related to the age and gender of participants. Moreover, following our results, Sheikhi et al. [27] revealed that the distance between the mental foramen and the inferior border of the mandible was statistically significant between males and females; they concluded that the MF anatomy is a valuable characteristic in dentulous and edentulous patients as well as in both genders. We did not see a significant effect for the variable age, perhaps in part, due to the rather narrow range of ages included in our study; all our patients were quite young and out of the puberty or decline periods which could better highlight the effect of age. Similar to the study by Sheikhi et al., we showed that the perpendicular distance to the inferior cortex of the mandible was significantly greater in long-face cases and was different between males and females. Such contradictory results may be due to the different populations studied, sample sizes, and methods in various studies. This calls for the assessment of the MF location using CBCTs in any intervention at or near the mental foramen level.

This study was limited by some factors. The sample size was not based on power calculations. However, it was much larger than the few similar studies in this regard. Furthermore, this study was retrospective; however, due to the biohazard of the X-ray, conducting prospective studies on human subjects and exposing humans to X-ray merely for the sake of research was impossible from an ethics standpoint. This limitation also applies to all similar radiographic studies; all such studies need to use radiographs that have been already taken for diagnostic and therapeutic purposes only. Since we had used CBCTs, it was better to also measure the size of the mental foramen; future CBCT studies should note this. As another limitation, the age range of patients entered in the study was narrow and only 10 years. This might be a reason why we did not observe any associations between age and the position of mental foramen. In this study, we averaged the measurements on the right and left sides. This resulted in more reliable outcomes compared to measuring only one side. On the other hand, it disallowed the comparison of the left and right sides in terms of their anatomic measurements. It is possible that some individuals have asymmetric mandibles and mental foramens.

## Conclusions

Sex dimorphism exists only in the case of the inferior-superior height of the mental foramen in the 3D space. The perpendicular distance between the foramen mental and the symphysis on the axial plane (the lateral “width” dimension) was the smallest in Class II and the longest in Class III cases. The changes observed in this variable between the 3 vertical growth patterns were smaller than those in different Classes, but still significant: this distance was the shortest in long faces and the largest in short faces. The perpendicular distance between the foramen mental and the inferior cortex of the mandible on the cross-sectional plane (the inferior-superior “height” dimension) was slightly but significantly larger in Class III cases than either of Class I or II patients; however, between Classes I and II there might not be a difference. This distance was the largest in long faces and smallest in short faces. The perpendicular distance between the foramen mental and the anterior border of the ramus on the parasagittal plane (the anteroposterior “length” dimension) was the largest in Class III and smallest in Class I. This dimension was slightly larger in short-face people than in normal-face or long-face individuals; the latter two might not differ in terms of this measurement.

## Data Availability

The data are available from the corresponding author upon request.

## References

[1] Fontenele RC, Farias Gomes A, Moreira NR, Costa ED, Oliveira ML, Freitas DQ (2022). Do the location and dimensions of the mental foramen differ among individuals of different facial types and skeletal classes? A CBCT study. J Prosthet Dentistry:S.

[2] Pelé A, Berry P-A, Evanno C, Jordana F. (2021) Evaluation of Mental Foramen with Cone Beam Computed Tomography: A Systematic Review of Literature. Radiology Research and Practice 2021:8897275. 10.1155/2021/8897275 PMC7806401 33505723

[3] Mashyakhy M, Mostafa A, Abeery A, Sairafi Z, Hakami N, Alroomy R et al. (2021) Structural Features of the Mental Foramen in a Saudi Subpopulation: A Retrospective CBCT Study. BioMed Research International 2021:1138675. 10.1155/2021/1138675 PMC8683170 34926680

[4] Lipski M, Tomaszewska I, Lipska W, Lis G, Tomaszewski K (2013). The mandible and its foramen: anatomy, anthropology, embryology and resulting clinical implications. Folia Morphol.

[5] Muinelo-Lorenzo J, Fernández-Alonso A, Smyth-Chamosa E, Suárez-Quintanilla JA, Varela-Mallou J, Suárez-Cunqueiro MM (2017). Predictive factors of the dimensions and location of mental foramen using cone beam computed tomography. PLoS ONE.

[6] Green RM. (1987) The position of the mental foramen: A comparison between the southern (Hong Kong) Chinese and other ethnic and racial groups. Oral Surgery, Oral Medicine, Oral Pathology 63:287–290. 10.1016/0030-4220(87)90191-5 3473355

[7] Chee NS, Park SJ, Son MH, Lee EJ, Lee SW (2014). Surgical Management of Edentulous Atrophic Mandible Fractures in the Elderly. Maxillofacial Plast Reconstr Surg.

[8] Zmyslowska-Polakowska E, Radwanski M, Ledzion S, Leski M, Zmyslowska A, Lukomska-Szymanska M. (2019) Evaluation of Size and Location of a Mental Foramen in the Polish Population Using Cone-Beam Computed Tomography. Biomed Res Int 2019:1659476. 10.1155/2019/1659476 PMC6334310 30719439

[9] Dos Santos Oliveira R, Rodrigues Coutinho M, Kühl Panzarella F (2018). Morphometric analysis of the Mental Foramen using Cone-Beam Computed Tomography. Int J Dent.

[10] Amini F, Alipanahi M, Rakhshan V, Shahab S, Niktash A (2017). Facial growth patterns and insertion sites of miniscrew implants. Implant Dent.

[11] Hwang S, Song J, Lee J, Choi YJ, Chung CJ, Kim KH (2018). Three-dimensional evaluation of dentofacial transverse widths in adults with different sagittal facial patterns. Am J Orthod Dentofac Orthop.

[12] Cartes G, Garay I, Deana NF, Navarro P, Alves N (2018). Mandibular Canal Course and the position of the Mental Foramen by panoramic X-Ray in Chilean individuals. Biomed Res Int.

[13] Saeed MS, Hagi FS (2019). Radiographic determination of mental foramen in patients with different skeletal occlusions in duhok governorate. J Duhok Univ.

[14] Shaban B, Khajavi A, Khaki N, Mohiti Y, Mehri T, Kermani H. (2017) Assessment of the anterior loop of the inferior alveolar nerve via cone-beam computed tomography. 43:395–400. 10.5125/jkaoms.2017.43.6.395 PMC5756796 29333369

[15] Srivastava KC (2021). A CBCT aided assessment for the location of mental foramen and the emergence pattern of mental nerve in different dentition status of the Saudi Arabian population. Brazilian Dent Sci.

[16] Naitoh M, Nakahara K, Suenaga Y, Gotoh K, Kondo S, Ariji E. (2010) Comparison between cone-beam and multislice computed tomography depicting mandibular neurovascular canal structures. Oral Surgery, Oral Medicine, Oral Pathology, Oral Radiology, and Endodontology 109:e25-e31. 10.1016/j.tripleo.2009.08.027 20123365

[17] Imada TSN, Fernandes LMPSR, Centurion BS, Oliveira-Santos Cd, Honório HM, Rubira-Bullen IRF (2014). Accessory mental foramina: prevalence, position and diameter assessed by cone-beam computed tomography and digital panoramic radiographs. Clin Oral Implants Res.

[18] Santini A, Land M (1990). A comparison of the position of the mental foramen in Chinese and British mandibles. Acta Anat (Basel).

[19] Sheikhi M, Karbasi Kheir M, Hekmatian E. (2015) Cone-Beam Computed Tomography Evaluation of Mental Foramen Variations: A Preliminary Study. Radiol Res Pract 2015:124635. 10.1155/2015/124635 PMC4644840 26609432

[20] Chen JC, Lin LM, Geist JR, Chen JY, Chen CH, Chen YK (2013). A retrospective comparison of the location and diameter of the inferior alveolar canal at the mental foramen and length of the anterior loop between American and Taiwanese cohorts using CBCT. Surg Radiol Anat.

[21] Khojastepour L, Mirbeigi S, Mirhadi S, Safaee A (2015). Location of Mental Foramen in a selected Iranian Population: a CBCT Assessment. Iran Endodontic J.

[22] Carruth P, He J, Benson BW, Schneiderman ED (2015). Analysis of the size and position of the Mental Foramen using the CS 9000 cone-beam computed Tomographic Unit. J Endod.

[23] Al-Mahalawy H, Al-Aithan H, Al-Kari B, Al-Jandan B, Shujaat S (2017). Determination of the position of mental foramen and frequency of anterior loop in Saudi population. A retrospective CBCT study. Saudi Dent J.

[24] Jacobson A, White L (2007). Radiographic cephalometry: from basics to 3-D imaging. Am J Orthod Dentofac Orthop.

[25] Velasco-Torres M, Padial-Molina M, Avila-Ortiz G, García-Delgado R, Catena A, Galindo-Moreno P. (2017) Inferior alveolar nerve trajectory, mental foramen location and incidence of mental nerve anterior loop. Medicina oral, patologia oral y cirugia bucal 22:e630. 10.4317/medoral.21905 PMC5694187 28809376

[26] Shahsavari S, Afsa M (2020). CBCT anatomic characteristics of Mental Foramen and Anterior Loop of Mandibular Canal in a population of South IRAN. J Dentomaxillofacial Radiol Pathol Surg.

[27] Sheikhi M, Kheir MK. (2016) CBCT Assessment of Mental Foramen Position Relative to Anatomical Landmarks. Int J Dent 2016:5821048. 10.1155/2016/5821048 PMC5141544 27999594

